# Keeping children healthy during and after COVID-19 pandemic: meeting youth physical activity needs

**DOI:** 10.1186/s12889-021-10545-x

**Published:** 2021-03-11

**Authors:** Andjelka Pavlovic, Laura F. DeFina, Breanna L. Natale, Shelby E. Thiele, Timothy J. Walker, Derek W. Craig, Georgina R. Vint, David Leonard, William L. Haskell, Harold W. Kohl

**Affiliations:** 1 The Cooper Institute, 12330 Preston Road, Dallas, TX 75230 USA; 2 Univeristy of Texas Health Science Center at Houston School of Public Health, Houston, TX USA; 3 Department of Cardiovascular Medicine, Stanford University, Palo Alto, California USA; 4 University of Texas at Austin, Austin, TX USA

**Keywords:** Physical education, Physical activity, Pandemic

## Abstract

**Background:**

The purpose of this study was to: 1) examine the maintenance of Physical Education and physical activity during the distance learning time, 2) determine the resources educators are utilizing to deliver PE curricula, and 3) understand the challenges experienced by educators during distance learning.

**Methods:**

A survey was sent to a cohort of school-based fitness assessment software users. Respondents were largely school-based individuals including PE teachers (*n* = 1789), school (*n* = 62) and district administrators (*n* = 64), nurses (*n* = 3), and “other” (*n* = 522).

**Results:**

Of 2440 respondents, most were from a city or suburb (69.7%), elementary or middle school (72.3%), and had Title 1 status (60.4%), an indicator of low socioeconomic status. Most campuses were closed during the COVID-19 pandemic (97.8%). Of the schools closed during the pandemic, only 2.8% had no prior PE requirements and that increased to 21% during the pandemic. In schools that remained open during the pandemic, 7.7% had no prior PE requirements and this increased to 60.5%. Importantly, 79% of respondents reported that students were either “significantly less” or “somewhat less” physically active during the closure. For closed schools, the most frequently cited challenges included “student access to online learning“, “teacher/student communication” and “teacher remote work arrangements”. For open schools, the most commonly reported challenges included “social distancing”, “access to gymnasium/equipment”, and “concern for personal health and wellbeing”.

**Conclusion:**

The COVID-19 pandemic has caused important reductions in PE requirements and time engaged in physical activity. Challenges experienced by teachers were identified for closed and open schools.

**Supplementary Information:**

The online version contains supplementary material available at 10.1186/s12889-021-10545-x.

## Background

In December 2019, a cluster of pneumonia like cases began to appear in China caused by a previously unknown virus, *SARS-CoV-2*. The known cases of disease caused by this virus (COVID-19) grew at an exponential rate causing the World Health Organization (WHO) to declare COVID-19 a pandemic in March 2020. By early September, approximately 25.6 million confirmed cases were diagnosed worldwide with over 850,000 deaths across more than 200 countries [10]. The pandemic nature of COVID-19 led to extraordinary societal measures aimed at containing the virus including closure of schools, social distancing, and sheltering in place at home across the United States (US) [6].

The impact of prolonged school closures on students undergoing “distance learning” for a pandemic is unknown. During extended breaks from school, such as summertime, there are significant challenges including “summer learning loss” which reflects not only stagnation of learned information but also regression. From an academic perspective, the loss is greater in spelling skills and math computation than reading [13]. These losses are more pronounced among children with low socioeconomic status, learning disabilities, and those who are English language learners [8].

In addition to academics, children’s health suffers during these breaks as body mass index (BMI) is known to increase during the summer as much as 5.2 percentile points in one study evaluating 3588 ethnically diverse children [7]. A smaller study evaluating the impact of break duration on weight showed that shorter breaks (3 and 7 weeks) mitigated increases in BMI versus 12-week breaks. Finally, children who participated in organized summer sports had significant improvement in their cardiovascular fitness compared to non-participants [1]. The current norm of distance learning, born out of necessity for social distancing, will likely have parallel effects. Given the known, negative impact of extended school breaks on youth health, it is critical to understand how current remote schooling affects physical activity levels of children.

Many schools and school districts are faced with the challenge of finding the best approaches to deliver distance education including Physical Education (PE). While instruction for basic subjects can be adapted for distance learning, less is known about PE given the space requirements for traditional lessons. Additionally, social distancing and sheltering at home may decrease opportunities for physical activity. Experts continue to emphasize the importance of a physically active lifestyle for health and stress management, assuming appropriate precautions [9]. The purpose of the current study was to: 1) examine the maintenance of PE and physical activity during the distance learning time, 2) determine the resources educators are utilizing to deliver PE curriculum, and 3) understand the challenges experienced by physical educators during distance learning.

## Methods

The “Meeting Children’s Physical Activity Needs during the COVID-19 Pandemic” questionnaire was developed to focus on three domains: 1) descriptive characteristics (job title of survey respondent, school location, school/district type and classification, and Title 1 status), 2) access to PE and physical activity, and 3) challenges associated with delivering PE and physical activity during the pandemic. To address these aims, the authors developed a new questionnaire as part of the present study and the list of all questions is included in the Supplement. In general, closed-ended response format was used to allow for efficient data aggregation and analysis. Further, emphasis was placed on limiting the number of questions for ease of administration while still capturing adequate information. The face validity was assessed by experts in the field. The questionnaire was then entered into Qualtrics XM® (SAP America Inc., Philadelphia, PA) and appropriate survey logic was inserted (i.e. PE questions were only displayed to PE teachers, etc.). The technical aspects of the survey tool were assessed by three experienced subject matter experts. Upon completion of the aforementioned assessment, the questionnaire was administered to potential participants.

The target population was selected from individuals with available email addresses within a single fitness assessment software (FitnessGram®), a tool to collect school-based fitness assessment data on children grades 3 to 12. This tool is widely used in the US and allowed for a diverse sample of respondents in terms of socioeconomics, regions, and states. Each survey participant answered a maximum of 15 questions based on their institution’s educational environment. FitnessGram users consist predominantly of PE teachers and school/district administrators including current or previous participants in The Cooper Institute Youth Programs (NFL Play 60 FitnessGram Project and the Healthy Zone™ School Program).

On April 23rd, 2020, the questionnaire was distributed to a convenience sample of 60,198 email contacts in the fitness assessment software contact database representing 29,786 active users and 30,412 prior users. Within the active contacts, 6839 schools were represented. The prevalence of viable and current email addresses in this database is unknown, making a definitive response rate unclear. In order to enhance response, four reminder emails were sent at approximately 2 day intervals and a giftcard raffle incentive was offered to participants. The deadline for completing the survey was May 4th, 2020 at 11:59 PM. A one-week response time was chosen at the guidance of school administration and to ensure availability of results well before fall planning. Ultimately, 3159 individuals responded to the survey. Of those, 594 individuals provided no answers and 125 provided partial demographic information, but answered no study-related questions. This resulted in a final analytic sample of 2440.

An introductory e-mail was sent to potential participants with study information, including the voluntary nature, and informed consent was implied when the respondent actively initiated the questionnaire. No identifying information was utilized in the study analysis. The research project and methodology were approved by The Cooper Institute Scientific and Institutional Review Boards.

Responses were exported from the Qualtrics XM® online survey platform and analyzed in SAS/STAT®, version 9.4 (SAS Institute, Inc. Cary NC USA). Descriptive statistics on respondent locations, job titles and school classification and status were tabulated along with selected responses on PE requirements and delivery before and during the pandemic. Changes in hours of PE required before and during the pandemic were tested using rank-sum statistics. The trend in responses across a Likert scale of accumulated physical activity changes among respondents whose school was closed was based on a model of log-linear counts. We considered each respondent as an independent observation, taking the view that respondents answered according to their own experiences, not as agents of a particular school or district.

## Results

Descriptive characteristics of respondents are presented in Table 1. The majority of respondents were PE teachers (*n* = 1789), followed by district (*n* = 64) and school (*n* = 62) administrators, and nurses (*n* = 3). An additional 522 survey respondents chose the “other” category, which consisted of classroom teachers, coaches, PE department chairs, athletic directors, and information technology personnel. The final sample of respondents had representation from each of the 50 US states, the District of Columbia and Puerto Rico. Additionally, 41 participants reported residing outside of the US. Overall, we saw little regional variation in the results. The greatest number of participants were from a city or suburb (69.7%), elementary or middle school (72.3%), and had Title 1 status, an indicator of low socioeconomic status, (60.4%). The vast majority of respondents reported their campus being closed during the COVID-19 pandemic (97.8%) with 97.4% of school closures occurring during the month of March 2020.

**Table 1:** Descriptive characteristics of survey respondents

Characteristic	N (%)
Job Title (n)	2440
- Physical Education Teacher	1789 (73.3)
- District Administrator	64 (2.6)
- School Administrator	62 (2.5)
- Nurse	3 (0.1)
- Other	522 (21.4)
School/District Type	2440
- City	896 (36.7)
- Suburb	806 (33)
- Town	385 (15.8)
- Rural	315 (12.9)
- I don’t know	38 (1.6)
School Classification	1846
- Elementary	809 (43.8)
- Middle	526 (28.5)
- High	369 (20)
- Other	142 (7.7)
Title 1 Status	1846
- Yes	1114 (60.4)
- No	614 (33.3)
- I don’t know	118 (6.4)
United States Regional Breakdown	2424
- Midwest	603 (24.7)
- Northeast	242 (9.9)
- South	1238 (50.7)
- West	314 (12.9)
Closed During the Pandemic	2432
- Yes	2379 (97.8)
- No	53 (2.2)
Closure Month	2340
- Prior to March 2020	15 (0.6)
- March 2020	2281 (97.4)
- April 2020	40 (1.7)
- May 2020	4 (0.2)

Survey participants were asked if their school had access to distance learning prior to the COVID-19 pandemic. The largest percentage of respondents reported either “no access” (19.7%) or “I don’t know” (18.9%) to the availability of distance learning. For those schools with a distance learning infrastructure, math and science were most commonly available at 19.5 and 17.4%, respectively. Health and PE lagged behind with 12.3 and 12.1% identifying these subjects as part of distance learning prior to the COVID-19 pandemic. For those schools that closed their campuses, 86.1% were reportedly able to deliver PE remotely. The preferred methods of delivering such PE curricula were via distance learning (47.7%), at-home resources (26.6%), and parent collaboration (15.3%).

**Fig. 1:**
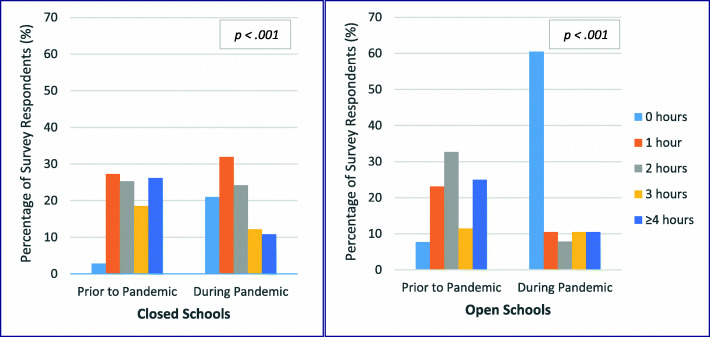
Physical Education requirements (hours/week) prior to versus during the COVID-19 pandemic based on school closure status (closed schools, left panel; open schools, right panel)

Prior to the COVID-19 pandemic, only 2.8 and 7.7% of pandemic closed and open schools, respectively, had no PE requirements (Fig. 1). The most common mandatory PE time was 1–2 h/week. During the pandemic, the percentage of schools with no PE requirement increased for both closed (21%) and open schools (60.5%). Furthermore, the total closed schools requiring at least 1 h of PE/week increased and the percentage of 3 and ≥ 4 required hours/week decreased. Overall, the survey results show that mandatory PE was maintained in the closed schools to a larger degree than open schools.

**Fig. 2:**
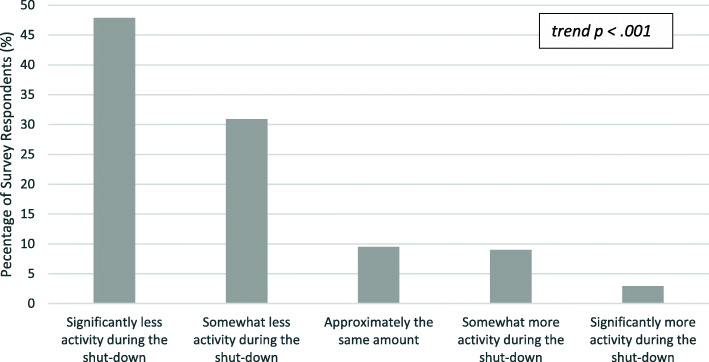
Survey respondent’s impression of the amount of physical activity accumulated during the school closure compared to the amount obtained in the typical school setting.

In addition to the PE related questions, we sought to determine the amount of physical activity and exercise obtained by students following the onset of the pandemic compared to the amount in a typical school day. Nearly 79% of survey respondents reported that students were accumulating either “significantly less” or “somewhat less” activity during the closure (Fig. 2). Respondents from Title 1 schools reported less physical activity for students during the shut-down than respondents from non-Title 1 schools or respondents unaware of their school’s Title 1 standing (*p* < 0.001). No significant differences in physical activity were observed across school level (i.e. elementary, middle, or high schools) or US regions. When able to deliver physical activity, the most commonly cited methods included at-home resources such as GoNoodle, OPEN, or YouTube (29%), e-learning platforms (27.9%), or virtual fitness/workouts by PE teachers (25.6%).

**Fig. 3:**
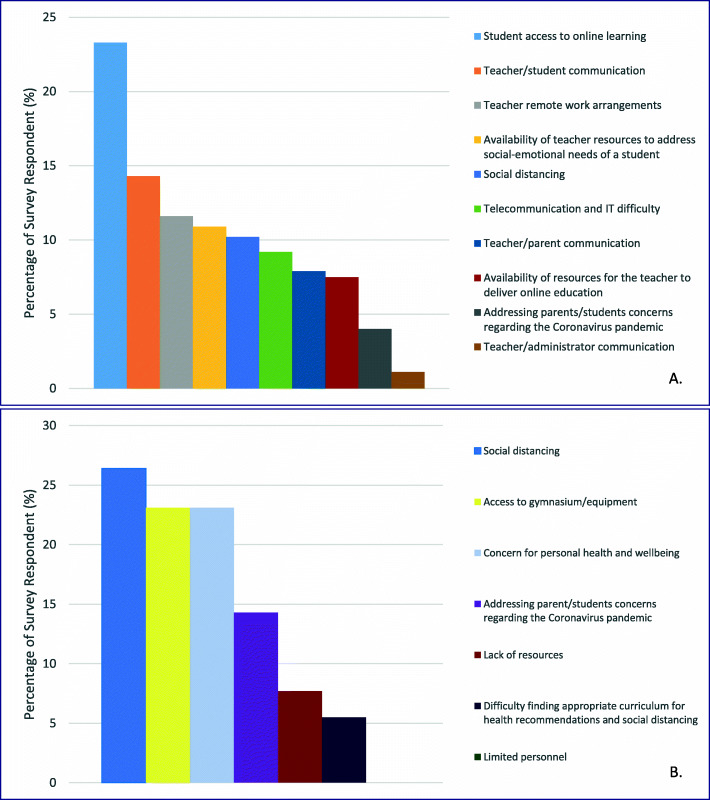
The survey respondents were asked to select the top three most significant challenges to teaching during the COVID-19 pandemic. **a** Challenges experienced by closed schools. **b** Challenges experiences by schools that remained open

Finally, the survey addressed the challenges experienced by teachers delivering PE in both closed (Fig. 3a) and open (Fig. 3b) schools. For schools closed during the pandemic, the three most frequently cited challenges included “student access to online learning” (23.3%), “teacher/student communication” (14.3%), and “teacher remote work arrangements” (11.6%). For school and district administrators, “availability of teacher resources to address social-emotional needs of a student” and “telecommunication and IT difficulty” were identified as substantial challenges. For schools remaining open throughout the COVID-19 pandemic, the most commonly reported challenges include “social distancing” (26.4%), “access to gymnasium/equipment” (23.1%), and “concern for personal health and wellbeing” (23.1%). In addition, the district administrators were concerned with “addressing parent/students concerns regarding the COVID-19 pandemic” (33.3%) (data not shown).

## Discussion

To date, this is the first study to examine the impact of the COVID-19 pandemic on PE and physical activity in youth as reported by a national convenience sample of physical educators, school/district administrators, and others. We showed that during the pandemic, respondents reported that youth across the US spent less time in both PE and physical activity. This finding, coupled with the dramatic relaxation of PE requirements, suggests a substantial reduction in opportunities for physical activity in youth during the pandemic. Although no other research regarding PE during a pandemic exists, previous literature has clearly demonstrated the negative impact of extended breaks from school on the health of youth [1, 2, 7]. For example, Carrel et al. [2] showed that in obese, middle-school children, a nine-month fitness-based PE intervention improved cardiorespiratory fitness, BMI, and insulin levels. Unfortunately, when the intervention was stopped during a 3-month summer break, all improvements were lost. This highlights the importance of ensuring continuation of PE and exercise during prolonged school breaks. Our survey discovered that a sizable percentage of respondents reported a reduction in or having no PE requirements during the pandemic. This “PE dumping” is likely multifaceted arising from acute health-related precautions, economic concerns, and the necessity to maintain academic programming.

The pandemic did not only impact PE, but also played a role in the amount of physical activity and exercise undertaken by youth across the nation. The results showed that 78.8% of survey participants believed their students were obtaining either “significantly less” or “somewhat less” physical activity compared to their typical school day. These findings demonstrate the need for at-home resources and solutions, so that youth can continue engaging in physical activity despite stay-at-home orders. Our findings were consistent with that of Pietrobelli et al. [11] who showed that children and adolescents, enrolled in a longitudinal observational study in Verona, Italy, decreased their time spent in sports by an average of 2.3 h/week and increased their screen time by approximately 4.9 h/week during the COVID-19 pandemic. Similar trends were observed in Shanghai, China among 2426 youth who participated in the Global Physical Activity Questionnaire developed by the WHO [15]. The authors found that the median time spent in physical activity decreased from 540 min/week to 105 min/week during the pandemic. Additionally, the prevalence of physically inactive youth increased from 21.3 to 65.6% during the same time period. Based on these findings, it is clear that opportunities for physical activity have decreased around the globe, and it is imperative that all youth are provided with safe, simple and easily implemented physical activity programs during the various phases of this pandemic [3].

The ramifications of decreased physical activity, school closures, and social isolation extend beyond declines in physical health. Mental health, specifically anxiety, stress, and depression, are negatively impacted when school-based resources are removed, leading to a worsening of these conditions [5]. As such, it is of utmost importance to support youth facing bereavement, parental unemployment, and drastic losses of household income. A possible method of improving mental health during the pandemic is to implement a structured exercise program that the child or adolescent finds enjoyable. Previous literature has shown that depressed adolescents treated with exercise were able to significantly reduce symptoms, improve psychosocial functioning, and maintain improvements at one-year follow up [4].

Finally, the findings of the present study identified and confirmed the substantial challenges experienced by teachers in both open and closed schools. While the concerns vary greatly between the two settings, this work provides great insight to educators and administrators for the upcoming school year. First, for schools that remained open, the PE curricular requirement was largely zero. This is not surprising as most of the US has been under social distancing orders, and as such, it is likely that schools removed activities where a student could break the “6 feet of separation” guideline and have to share exercise and/or sports equipment. For the schools that remained open, the biggest challenges were social distancing, access to a gymnasium, and concern for personal health and wellbeing. These challenges are especially important, as they should be utilized to inform educators and administrators returning to campus in the future. It will be critical to identify curriculum, activities, and equipment that allow youth to be physically active while avoiding close contact or use of uncleaned equipment.

In the closed schools, the most frequent challenge experienced by teachers as well as school/district administrators during the pandemic was “student access to online learning.” Considering that 60.4% of the respondents were from Title 1 schools, it is possible that the resources to carry out virtual learning were not immediately available to a large percentage of students. It was reassuring to see that an emphasis was put on having at least 1 h/week of PE in the at-home environment, which supports expert guidelines that everyone should remain physically active with appropriate precautions. Nonetheless, the general impression from the respondents was that there was a moderate to significant decrease in physical activity. This may be driven by economic, social, and environmental factors that prevent children from engaging in physical activity. If the pandemic continues, it will be critical to ensure that all children have access to physical activity motivating materials.

A strength of this study is the inclusion of a national convenience sample of survey respondents across each of the 50 US states, including the District of Columbia and Puerto Rico. An important benefit of this study was that 60.4% of the respondents were from Title 1 schools, which is comparable to the reported 2015–2016 national average [12]. Another strength of the study was the ability to gather information from various sources within the educational hierarchy including but not limited to physical educators, district and school administrators, athletic directors, grade teachers, coaches and nurses. The individually reported data reflect the unique respondent’s personal experience of their school environment and curriculum. Additionally, data were collected in a limited time frame after most schools have been working remotely for at least a month, and in general, were unaware of plans for returning to school. Hence, the accuracy of the perceived experiences was likely high. A limitation of the study is the lack of a scientifically validated questionnaire for school-level information that would answer the proposed aims. However, face validity was considered. Another limitation of the study is that a single survey respondent answered the questions based on their perception of the school environment, which could lead to biased responses. However, most participants who completed the survey were physical educators and thus the local expert on the topic. While the findings for open schools provide valuable insight, these results should be interpreted with caution as only a small number of schools in our study cohort remained open during the early phase of the COVID-19 pandemic. Lastly, data collected on students’ physical activity levels during the pandemic were based on subjective responses by the teacher rather than an objective tool such as an accelerometer.

## Conclusion

In conclusion, the study showed the impact of COVID-19 on PE and physical activity across the nation. Significant reductions were observed in requirements for PE as well as the amount of time engaged in physical activity. Both of these factors could lead to higher obesity and lower cardiorespiratory fitness among children, predisposing them to cardiometabolic disease and other health consequences. Additionally, the most significant challenges experienced by teachers during the pandemic were identified for both closed and open schools. These results clearly support that different measures must be taken to keep children active and fit based on their educational environment. Schools should continue to maintain a PE requirement as a critical educational component during the pandemic. For those schooling remotely, it is critical to prioritize online learning options in the school curriculum and provide access to physical activity promoting materials. A potential obstacle, particularly in Title 1 schools, is the availability of adults to assist in the remote education as parents may need to work. It will likely require a combination of school- and community-based leadership support to ensure that children have adult supervision for both academic and physical education. For those attending school, the issues are more complicated as activity must be integrated with the need for social distancing. Optimally, PE curriculum with outdoor activities and without shareable equipment would provide opportunity for PE and exercise. Future research should examine the optimal methods to teach in both environments considering current acute health issues, or Coronavirus, and long-term obesity and inactivity related concerns such as diabetes. Additionally, future work should focus on developing carefully crafted questionnaires that will provide optimal insight for longitudinal and post-COVID-19 studies. It must be emphasized that maintenance of physical education and activity needs to be highly prioritized in the school or district planning as a healthy child is known to be a better learner with less behavioral issues [14].

## Supplementary Information

**Additional file 1.**

## Data Availability

The datasets during and/or analyzed during the current study available from the corresponding author on reasonable request.

## Abbreviations

BMI: Body Mass Index
PE: Physical Education
US: United States
WHO: World Health Organization
