# Supplementary material for: Keeping children healthy during and after COVID-19 pandemic: meeting youth physical activity needs
Source: BMC Public Health. 2021 Mar 11;21:485. doi: 10.1186/s12889-021-10545-x (PMC7948663; doi:10.1186/s12889-021-10545-x)
Supplement: Supplementary file 1 — Additional file 1. [file 12889_2021_10545_MOESM1_ESM.pdf]

Meeting children's physical activity needs during the Coronavirus pandemic

Demographic Information (answered by all participants)

1. Job Title
    a. Physical Education Teacher
    b. Nurse
    c. School Administrator
    d. District Administrator
    e. Other: ____
2. State
    - State Dropdown
3. School/District type
    a. City (inside a city with a large population)
    b. Suburb (outside of the city with a large population)
    c. Town (self-contained settlement, all aspects of a large city, but smaller)
    d. Rural (low population density, open swath of land)
    e. I don't know
4. School classification (n/a district administrator)
    a. Elementary School
    b. Middle School
    c. High School
    d. Other: _________
5. Is your school a Title 1 school (at least 40% of students on free/reduced lunch)? (n/a district administrator)
    a. Yes
    b. No
    c. I don't know
6. Is the physical location of your school/district closed to students during the current Coronavirus pandemic?
    a. Yes
    b. No

All Schools:

7. Are you, or the appropriate teacher within your school/district, able to deliver Physical Education to your students during the Coronavirus pandemic?
    a. Yes
    b. No
    c. I don't know
8. What is <u>*your impression*</u> of the amount of physical activity accumulated following the school closure as compared to the amount obtained in a typical school setting?
    a. Significantly more activity during the shut-down

b. Somewhat more activity during the shut-down
c. Approximately the same amount
d. Somewhat less activity during the shut-down
e. Significantly less activity during the shut-down

Closed Schools:

9. If yes, when did your school/district close?
    - Mm/dd/yyyy
10. Prior to the Coronavirus pandemic, how many hours per week were students required to engage in Physical Education class on campus?
    a. 0 hours
    b. 1 hour
    c. 2 hours
    d. 3 hours
    e. 4 or more hours
11. From the list below, did your school have access to the following distance learning prior to the Coronavirus pandemic? Select all that apply.
    a. Math
    b. Science
    c. Health
    d. Physical Education
    e. We did not have access
    f. I don't know
12. Currently, how many hours per week are students required to engage in Physical Education?
    a. 0 hours
    b. 1 hour
    c. 2 hours
    d. 3 hours
    e. 4 or more hours
13. What is your school/district's current method of delivering a structured Physical Education curriculum? Select all that apply.
    a. Distance learning
    b. At home resources (i.e. CATCH, SPARK, social media, etc.)
    c. FitnessGram SmartCoach Resources
    d. Parent collaboration (i.e. assignments delivered to parents via conference calls or email)
    e. Other: _______
14. From the list below, select what you believe to be the top 3 most significant challenges during the Coronavirus pandemic. Select 3 items.
    a. Teacher remote work arrangements
    b. Availability of resources for the teacher to deliver online education
    c. Student access to online learning
    d. Teacher/parent communication
    e. Teacher/administrator communication
    f. Teacher/student communication

    g. Availability of teacher resources to address social-emotional needs of a student
    h. Addressing parents/students concerns regarding the Coronavirus pandemic
    i. Social distancing
    j. Telecommunication and IT difficulty
    k. None of the above
15. During school closure, what is your school/district's method of engaging students in physical activity? Select all that apply.
    a. E-learning platform
    b. At home resources (i.e. OPEN module, GoNoodle, AHA Virtual Experiences, YouTube, etc.)
    c. Virtual fitness/workout sessions by Physical Education teachers
    d. Parent collaboration
    e. Other: ___

Open Schools only:

16. Currently, how many hours per week are students required to engage in Physical Education class on campus?
    a. 0 hours
    b. 1 hour
    c. 2 hours
    d. 3 hours
    e. 4 or more hours
17. From the list below, select what you believe to be the top 3 most significant challenges implementing on-campus Physical Education during the Coronavirus pandemic. Select 3 items.
    a. Lack of resources
    b. Limited personnel
    c. Access to gymnasium/equipment
    d. Difficulty finding appropriate curriculum for health recommendations and social distancing
    e. Addressing parents/students concerns regarding the Coronavirus pandemic
    f. Social distancing
    g. Concern of personal health and well being
